# Cold-Plasma Method in Counteracting Prosthetic Stomatitis: Analysis of the Influence of Cold Plasma on Prosthetic Materials

**DOI:** 10.3390/ma18174162

**Published:** 2025-09-04

**Authors:** Agnieszka Mazur-Lesz, Joanna Pawłat, Piotr Terebun, Dawid Zarzeczny, Elżbieta Grządka, Agnieszka Starek-Wójcicka, Michał Kwiatkowski, Irena Malinowska, Magdalena Mnichowska-Polanowska, Monika Machoy

**Affiliations:** 1 Private Dental Office, Witkiewicza 49u/14 Street, 71-124 Szczecin, Poland; 2 Department of Electrical Engineering and Smart Technologies, Lublin University of Technology, Nadbystrzycka Street 38A, 20-618 Lublin, Poland; j.pawlat@pollub.pl (J.P.); p.terebun@pollub.pl (P.T.); d.zarzeczny@pollub.pl (D.Z.); m.kwiatkowski@pollub.pl (M.K.); 3 Faculty of Chemistry, Institute of Chemical Sciences, Maria Curie-Skłodowska University, M. Skłodowskiej-Curie 3 Sq., 20-031 Lublin, Poland; irena.malinowska@mail.umcs.pl; 4 Department of Biological Bases of Food and Feed Technologies, Faculty of Production Engineering, University of Life Sciences in Lublin, 20-612 Lublin, Poland; agnieszka.starek@up.lublin.pl; 5 Department of Microbiology, Immunology and Laboratory Medicine, Pomeranian Medical University, Powstańców Wielkopolskich 72 Street, SPSK 2, 70-111 Szczecin, Poland; magdalena.mnichowska.polanowska@pum.edu.pl; 6 Department of Periodontology, Faculty of Medicine and Dentistry, Pomeranian Medical University, Powstańców Wielkopolskich 72 Street, SPSK 2, 70-111 Szczecin, Poland; monika.machoy@pum.edu.pl

**Keywords:** cold atmospheric plasma, denture stomatitis, *Candida albicans*, *Candida glabrata*, acryl, acetal, prosthetic metal alloy

## Abstract

The aim of this study was to determine the possibilities of using cold-plasma technology in counteracting the development of denture stomatitis (DS) in patients using different kinds of prosthetic restorations. The study focused mainly on the effect of cold atmospheric plasma on prosthetic materials, such as acryl (AR), acetal (AT), and a prosthetic metal alloy (MA). The materials were tested in terms of the effect of the plasma exposure time (5, 10, and 20 min) on changes in the chemical composition, morphology, and surface topography (FT-IR, SEM-EDS, optical profilometer) as well as changes in the color and contact angle (spectrophotometer, goniometer) after the plasma process. Furthermore, the ability of reference fungi (*C. albicans* and *C. glabrata*) to adhere to non-modified and cold atmospheric plasma (CAP)-modified dental materials was examined to evaluate the susceptibility of dental material surfaces to 12 h fungal contamination. The obtained results demonstrate that CAP appears viable for the surface modification of the acetal resin and the metal alloy, not compromising their structural integrity while variably limiting fungal overgrowth involved in the development of DS, whereas its application to the acrylic resin may be inadvisable due to morphological and optical alterations.

## 1. Introduction

According to the World Health Organization (WHO), severe tooth loss, recognized as a disability, affects over 250 million people worldwide [1]. One of the most common means of oral rehabilitation are removable dentures, which, due to relatively low costs and government funding in many European countries, remain accessible to a broad patient population. Removable dentures play a key role in restoring both esthetics and masticatory function. The most widely used materials for their fabrication are acrylic resin, acetal, and cobalt–chromium–molybdenum (Co-Cr-Mo) alloys.

Acrylic resin (AR), based on methyl methacrylate, is typically available in powder or liquid form and can be polymerized thermally, chemically, or using light [2]. Despite its popularity, AR is prone to rapid absorption, which favors fungal colonization, lowers denture stability, and increases the risk of inflammation, discoloration, and allergic reactions [3,4]. Acetal (AT), or polyoxymethylene, offers superior esthetics, biocompatibility, chemical stability, and comfort compared with acrylics, while showing reduced adhesion of *Candida* spp. [5,6,7,8]. However, AT is more fracture-prone than metal frameworks. The Co-Cr-Mo alloy, widely used in skeletal dentures and partial denture elements, provides high strength, rigidity, corrosion resistance, and good casting fluidity [9,10,11,12,13].

However, using a dental prosthesis carries the risk of denture stomatitis (DS). DS, primarily associated with *Candida* spp., affects 15–70% of denture wearers [14,15]. It is a recurrent local infection, often necessitating new prostheses. Conventional therapies remain insufficient, which emphasizes the importance of alternative methods such as cold atmospheric plasma (CAP) treatment. The antimicrobial effect of CAP was first described in the late 1990s [16]. This method generates a mixture of reactive oxygen and nitrogen species (ROS/RNS), UV radiation, and electric fields, which effectively damage microbial cells and biofilm matrices, leading to significant reductions in viable bacteria and fungi on prosthetic and implant materials. Moreover, it does not damage surrounding healthy tissues [17], and its ability to limit antibiotic use makes it a promising tool against microbial resistance [18]. In dentistry, CAP has been investigated for periodontitis and peri-implantitis therapy, caries management, bleaching, endodontics, and biofilm decontamination [19,20,21]. Numerous reports demonstrate that CAP exhibits antifungal efficacy against *Candida albicans* on denture materials, particularly acrylic resin [22,23,24,25,26,27]. Delben et al. showed that CAP substantially decreased colony-forming units (CFU) of *Candida albicans* and *Staphylococcus aureus* while causing no toxic effects on in vitro reconstructed oral epithelium, confirming the biological safety of short CAP exposures [28]. Laboratory and clinical studies on dentures demonstrated that CAP reduces early *C. albicans* adherence to acrylic surfaces and increases hydrophilicity without major chemical degradation of the material in most protocols, although its efficacy against older, mature biofilms remains limited; for example, it was highly effective against 2-day biofilms but exerted little impact on 7–16-day biofilms [29]. It is also known that CAP increases surface hydrophilicity and reduces early fungal adherence without significantly altering the chemical structure of AR, although some studies suggest possible changes [25,26]. While CAP frequently increases surface roughness, this does not appear to promote fungal adhesion [24]. In endodontics, systematic reviews and meta-analyses highlight the strong potential of CAP in root canal disinfection, particularly against *Enterococcus faecalis*, although its efficacy depends on the device parameters, carrier gas, exposure time, and application distance; standardized protocols are required before clinical implementation [20]. In the case of implants and titanium surfaces, studies conducted by Kamionka et al. demonstrated that CAP can effectively remove biofilms and may enhance host cell adhesion after decontamination, suggesting its promising role in peri-implantitis and periodontal therapy [30]. A systematic review compiled by Jungbauer et al. [31] summarized robust in vitro evidence of broad antimicrobial activity of CAP against oral pathogens but emphasized methodological heterogeneity and the need for further clinical trials.

Simultaneous studies on the effect of CAP on three different types of prosthetic materials (acryl, acetal, and metal alloy) conducted so far have not analyzed the use of metal alloys or presented contradictory results with respect to acryl. Moreover, the use of different types of cold plasma and different plasma treatment times significantly complicates the comparison of the effect of plasma on different prosthetic materials. It is also hypothesized that cold-plasma treatment improves the hygienic conditions of oral tissues and dental prosthetics by impairing fungal adhesion and biofilm formation, i.e., processes involved in the development of DS.

Based on the above findings, the aim of this study was to evaluate the effect of cold atmospheric plasma (CAP) with helium and oxygen on three prosthetic materials: acrylic resin, acetal resin, and a metal alloy (Co-Cr-Mo) at distinct exposure times. Specifically, we analyzed changes in their chemical composition, surface morphology, surface roughness, contact angle, and color as well as the adhesion of *Candida albicans* ATCC 10231 and *Candida glabrata* ATCC 90030 fungi.

## 2. Materials and Methods

### 2.1. Materials

The research material comprised acrylic and acetal plates (5 × 5 × 2 mm) and disks (Ø5 × 1 mm) cut from a prosthetic metal alloy. These sample dimensions and shapes were selected to ensure adequate mechanical strength, compatibility with the measuring apparatus, and reproducibility of surface preparation. Four samples were prepared for each test type.

#### 2.1.1. Acrylic Resin

Polymerization, a standard dental laboratory procedure, requires precise control to ensure optimal material properties [32]. In this study, polymethyl methacrylate (PMMA) powder was mixed with methyl methacrylate (MMA) monomer to obtain acrylic dough for denture base fabrication. The process proceeded through the characteristic stages: wet sand, unsticking, thread, dough, and polymerization, followed by the typical reaction phases of initiation, propagation, termination, and chain transfer. After thermal polymerization, the material was processed in a dental laboratory and cut into acrylic plates (5 × 5 × 2 mm). The acrylic resin used was Villacryl H Plus^®^ (Zhermack, Badia Polesine, Italy), with the chemical composition given in Table 1.

#### 2.1.2. Acetal Resin

Acetal resin, supplied as a finished product and virtually free of residual monomers, was thermally conditioned in an injection molding machine prior to prosthetic application. The sample fabrication required strict control of time, pressure, and temperature during all stages of injection and cooling. The process parameters were as follows: melting point 220 °C, melting time 20 min, application time 2–5 min, cooling time 20–40 min, and application pressure 4–6 bar. Samples were then prepared in a dental laboratory following the same procedures as for acrylic. The material used was Dental D^®^ acetal resin (Pressing Dental, Falciano, San Marino), with the chemical composition provided in Table 1.

#### 2.1.3. Metal Alloy

In this study, metal-alloy blocks were processed with CAD/CAM and cut into disks (Ø5 × 2 mm). The alloy used was Biosil F^®^ (DeguDent, Hanau, Germany), with the chemical composition shown in Table 1.

### 2.2. Methods

#### 2.2.1. Cold Atmospheric Plasma Treatment

**Figure 1:**
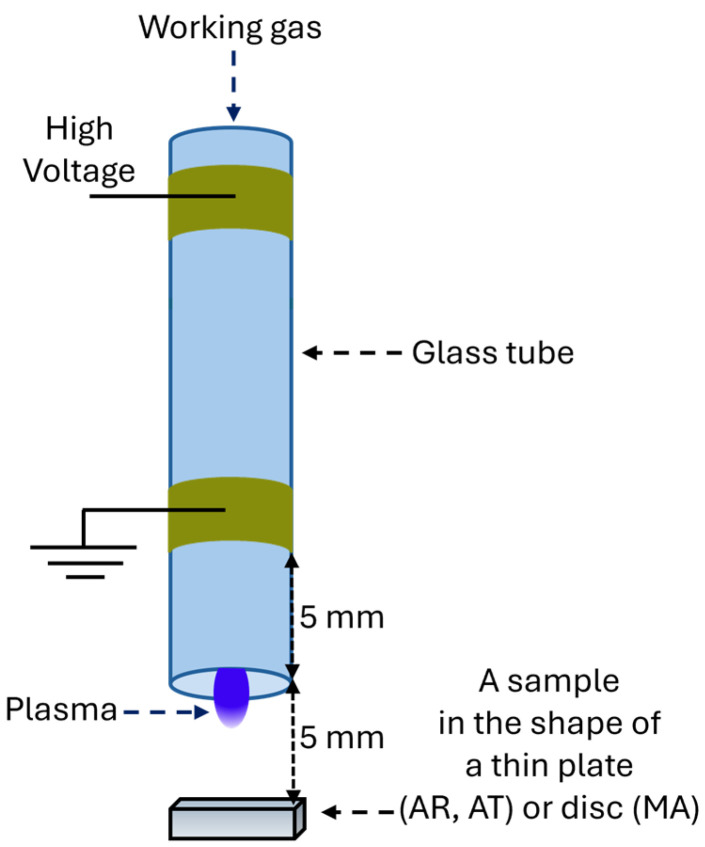
Scheme of the plasma processing system.

The samples were treated with an atmospheric pressure plasma jet generated in a reactor consisting of a 5 mm glass tube with two ring electrodes spaced 10 mm apart (Figure 1). Plasma formed inside the nozzle was transported by forced gas flow to the sample positioned 5 mm from the nozzle tip. The working gas was helium (purity 4.0, 1667 sccm) with a small oxygen admixture (purity 5.0, 13 sccm), where helium facilitated discharge ignition. The reactor was powered by a 28.5 kHz VLF signal at 3.25 kV RMS and 10 W. The treatment times were 5, 10, and 20 min, and the sample surface temperature, measured with a K-type thermocouple, did not exceed 32.6 °C.

#### 2.2.2. FT-IR

The FT-IR analysis was conducted using a FT-IR-4200 type A spectrometer (JASCO Corporation, Tokyo, Japan) with a ATR PRO ONE accessory (JASCO Corporation, Tokyo, Japan) equipped with a diamond crystal (Jasco, Tokyo, Japan). Spectra were recorded in the 4000–500 cm^−1^ range, at 4 cm^−1^ resolution, and averaged over 32 scans.

#### 2.2.3. SEM-EDS

Morphological changes after plasma treatment (air plasma, 5, 10, or 20 min) and the elemental composition (EDS) were analyzed using a scanning electron microscope (SEM, Quanta 3D FEG, FEI, Hillsboro, OR, USA) operated at 5 kV. Micrographs were acquired at 500× and 5000× magnifications. Quantitative results represent the mean of four replicates with standard deviations.

#### 2.2.4. Surface Roughness

Surface topography was analyzed using an optical profilometer (Contour GT-K1, Bruker, Ettlingen Germany) based on light interference and fringe imaging. Three-dimensional surface images were acquired to determine roughness parameters (R_a_, R_q_, R_t_, R_v_). Measurements were taken at three sites within a 58 × 44 μm^2^ area per sample, averaged, and compared before and after plasma treatment at identical locations to minimize natural variability. Data analysis was performed with Veeco Vision 4.20 software (Veeco Instruments Inc., Plainview, New York, USA).

#### 2.2.5. Contact Angle

The contact angle was measured using a DSA25E goniometer (KRÜSS, Hamburg, Germany) applying the sessile drop method. For each condition, 3 contact angle measurements were taken (one at the center of the sample and two at a distance of 10 mm), each time placing 0.5 µL of distilled water. In the case of plasma processing, measurements were performed 10 s after each treatment. The contact angle was determined in the KRÜSS Advance software, version 1.3.1.0 (KRÜSS GmbH, Hamburg, Germany) using the ellipse (tangent − 1) fitting method.

#### 2.2.6. Color Measurements

Color measurements were performed with an SV-100 spectrophotometer (Tri-Color, Narama, Poland) (aperture 3 × 1 mm) in the CIE Lab color space. The device was calibrated with a white standard prior to testing. Measurements were carried out under D65 illumination with d/8° geometry, at multiple points on each sample surface. Recorded parameters included L* (lightness), a* (red–green), and b* (blue–yellow).

#### 2.2.7. The 12 h Adhesion of Single-Species *Candida albicans* and *Candida glabrata* to Dental Materials

*Candida albicans* (ATCC 10231) and *Candida glabrata* (ATCC 90030) were cultured on Sabouraud dextrose agar (SDA;, bioMérieux Polska, Warsaw, Poland) at 30 °C for 24 h. Inocula were prepared in Sabouraud dextrose broth (SDB;bioMérieux Polska, Warsaw, Poland) and adjusted to 0.5 McFarland standard, corresponding to 1 × 10^6^ CFU/mL for *C. albicans* and 5 × 10^6^ CFU/mL for *C. glabrata*, confirmed by serial dilutions. Dental material samples (AR, AT, MA) were immersed in standardized suspensions. For each material, unexposed controls and CAP-treated samples (5, 10, 20 min) were tested for *Candida* early biofilm formation, (measured as 12 h adhesion). The 12 h adhesion of the single-species *C. albicans* ATCC 101231 and *C. glabrata* ATCC 90030 to three CAP-treated prosthetic materials, each subjected to three distinct CAP exposure times in salivary pellicle-free conditions, was evaluated. The study assessed *C. albicans* adhesion at the 12 h time point, because hallmark structural components of early biofilm were present. *C. glabrata* adhesion was also measured at the 12 h time point to compare it with that of *C. albicans.* After 12 h incubation at 30 °C, the samples were washed five times in phosphate-buffered saline (PBS) to remove non-adherent cells. Adherent cells were detached using a vortex–sonication–vortex protocol (30 s vortex, 45 s sonication at 30 kHz, room temperature, followed by 30 s vortex) as described previously [33]. The suspensions were serially diluted (1:100, 1:1000, 1:10,000), and 100 µL were plated on SDA for CFU determination. Colonies were manually counted Scan^®^100, (Interscience, Saint-Nom-la-Breteche, France); eCount™ colony counter pen (Heathrow Scientific, Vernon Hills, IL, USA), and the results were expressed as CFU/mm^2^. The reduction in *C. albicans* and *C. glabrata* growth on the CAP-exposed samples was calculated relative to the controls. Each material was tested in triplicate in four conditions (three CAP exposures and one control).

## 3. Results and Discussion

### 3.1. Influence of CAP on Physico-Chemical Properties of Prosthetic Materials

#### 3.1.1. Changes in the Chemical Composition of the CAP-Treated Surfaces of the Prosthetic Materials

Table 1 presents the analysis of the surface composition of the tested prosthetic materials before the plasma procedure and after 5, 10, and 20 min of cold-plasma exposure of the surface. The first of the tested materials, acryl (AR), is a material based on the methyl ester of methacrylic acid, i.e., methyl methacrylate (monomer). According to the obtained SEM-EDS data, the tested acrylic consisted of about 65% carbon and about 35% oxygen. Moreover, its composition did not change as a result of the cold-plasma action regardless of the duration of the procedure, which allowed us to conclude that this material was chemically resistant to the action of CAP. The second of the tested materials, acetal, is chemically a product of formaldehyde polymerization. This material consisted of approximately 47% carbon and 53% oxygen. The plasma treatment did not change the chemical composition of the material regardless of the treatment time. The third type of samples was a prosthetic metal alloy consisting mainly of cobalt (about 50%) and chromium (about 24%) and containing admixtures of other chemical elements, such as oxygen, carbon, molybdenum, silicon, aluminum, and iron. In this case, the plasma treatment resulted in insignificant changes in the chemical composition regardless of the time. Based on the above findings, it can be concluded that cold-plasma treatment is chemically safe for use with prosthetic materials, as it does not adversely affect their composition.

**Figure 2:**
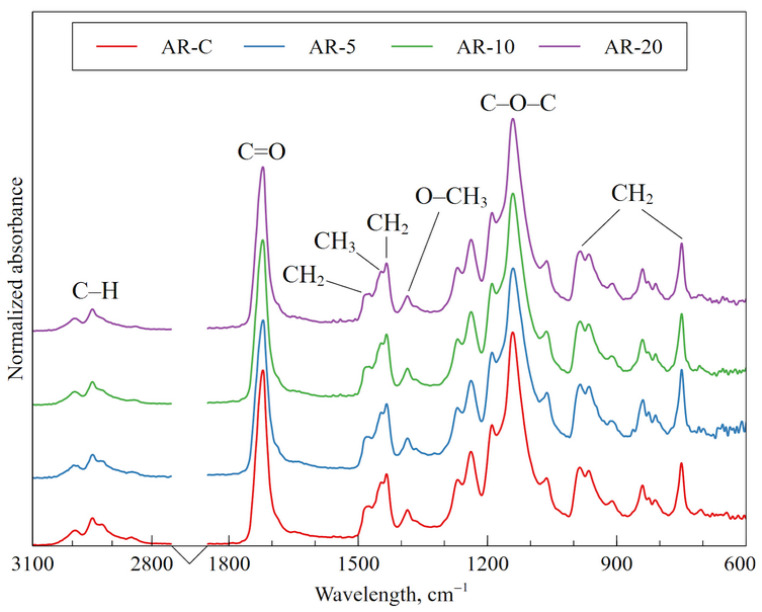
Normalized FT-IR spectra of AR samples for different plasma treatment times.

The normalized FT-IR spectra of the AR samples are shown in Figure 2, where the wavelength axis has been clipped to areas depicting characteristic bands for better readability. The obtained spectra are similar to those obtained by other authors studying acrylic materials [34,35,36,37]. The band at 2950 cm^−1^ can be correlated with the characteristic methylene C–H stretch bands. The ester group can be seen as intense carbonyl C=O stretching vibrations occurring at 1722 cm^−1^. The asymmetric stretching of CH_2_ is shown by two bands at 1478 cm^−1^ and 1437 cm^−1^, while the CH_3_ is visible at the 1444 cm^−1^ band. The band visible at 1386 cm^−1^ can be attributed to the O–CH_3_ deformation and the asymmetric peak at 1144 cm^−1^ can be assigned to the C-O-C group. The other CH_2_ vibrations can be associated with the bands at 995 cm^−1^ and 750 cm^−1^. No changes were observed in the spectra in any of the treatment times tested, which could indicate that no new chemical compounds or functional groups were formed at the penetration depth tested. Comparing this with analyses performed using other methods, which showed a change in the contact angle and color, it can be assumed that changes induced by the plasma treatment occur only in a thin layer near the surface of the tested materials. The results may also have been influenced by the shape of the spectra of the untreated samples, which overlap with the bands of potential new functional groups formed after the treatment in air, such as the C=O bands for wavelengths of 1740–1710 cm^−1^, or their low intensity.

**Figure 3:**
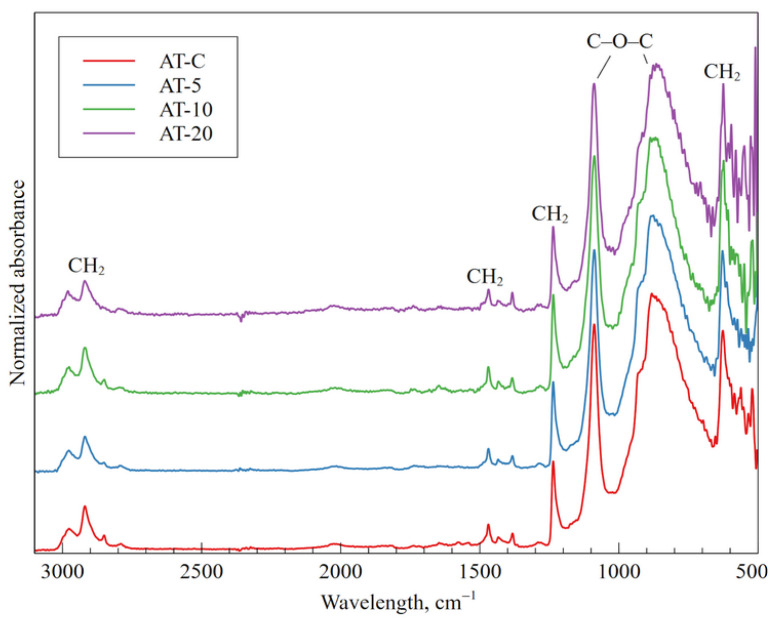
Normalized FT-IR spectra of AT samples for different plasma treatment times.

A comparison of normalized FT-IR spectra of AT is shown in Figure 3. Four bands at wavelengths 2920 cm^−1^ [38], 1468 cm^−1^ [39], 1235 cm^−1^ [38], and 625 cm^−1^ [38] can be related to the CH_2_ stretching [38,39]. The band at wavelength 1235 cm^−1^ can also be related to the C-O-C stretching which is observed for the band at a wavelength of 1088 cm^−1^ and a broad band around 900 cm^−1^ [40]. As before, no significant changes were observed after the plasma treatment.

#### 3.1.2. Changes in the Morphology of the CAP-Treated Surfaces of the Prosthetic Materials

**Figure 4:**
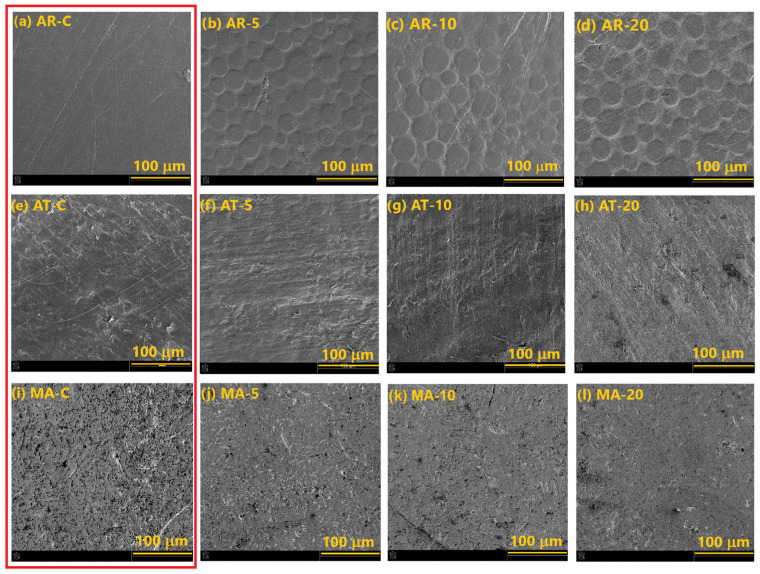
SEM micrographs showing the effect of the plasma treatment time (5, 10, 20 min) on the prosthetic materials: acryl (AR), acetal (AT), and prosthetic alloy (MA), C-control samples (in red square): (**a**) AR-C; (**b**) AR-5; (**c**) AR-10; (**d**) AR-20; (**e**) AT-C; (**f**) AT-5; (**g**) AT-10; (**h**) AT-20; (**i**) MA-C; (**j**) MA-5; (**k**) MA-10; (**l**) MA-20.

Figure 4 shows the effect of the plasma treatment time on the surface morphology of the tested prosthetic materials. In the case of the acrylic materials, the CAP treatment caused significant surface changes, which were more visible at the longer CAP treatment time. As can be seen, the surface lost its homogeneity. There are visible inhomogeneities of the round shape on the surface, which are deeper and more distinct with the extended plasma exposure. These findings suggest that the plasma treatment altered the acrylic surface, underscoring the importance of optimizing the exposure time to ensure microbiological efficacy without significantly impairing the structural integrity or durability of the material. In the case of the tested acetal and prosthetic alloy, no morphological changes were observed after the plasma treatment. This proves that cold plasma can be successfully used to process these materials without the risk of deterioration of their morphological properties and therefore their strength and service life.

**Figure 5:**
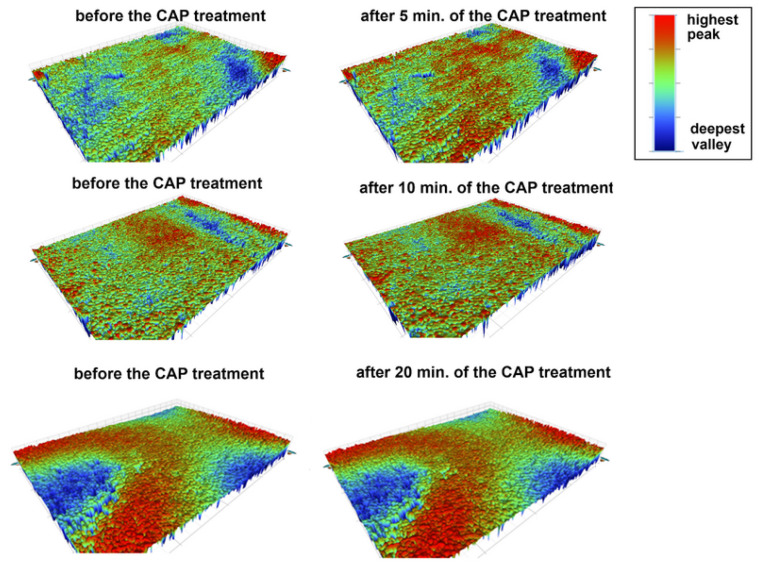
Images of the surface of acryl (AR) before and after the CAP treatment (5, 10, 20 min), C-control/untreated sample (the colors represent the topography of the surface; the higher the peaks—the more red, the deepest the valleys—the more navy blue).

**Figure 6:**
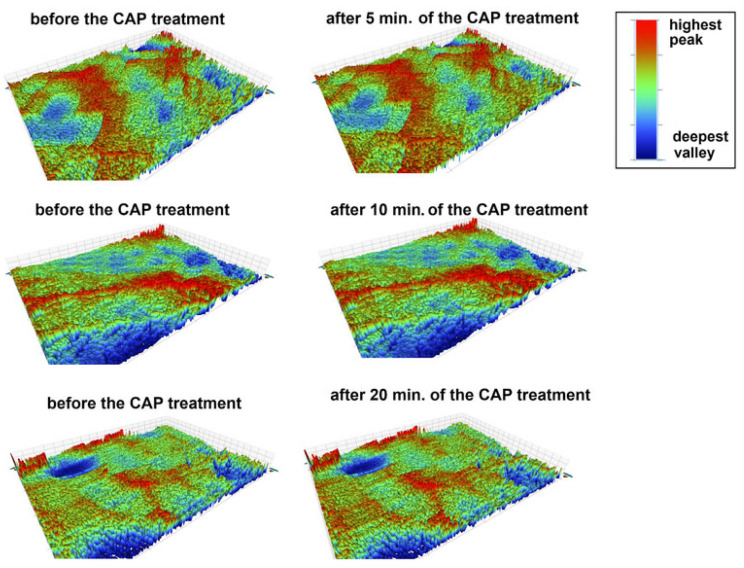
Images of the surface of acetal (AT) before and after the CAP treatment (5, 10, 20 min), C-control/untreated sample (the colors represent the topography of the surface; the higher the peaks—the more red, the deepest the valleys—the more navy blue).

**Figure 7:**
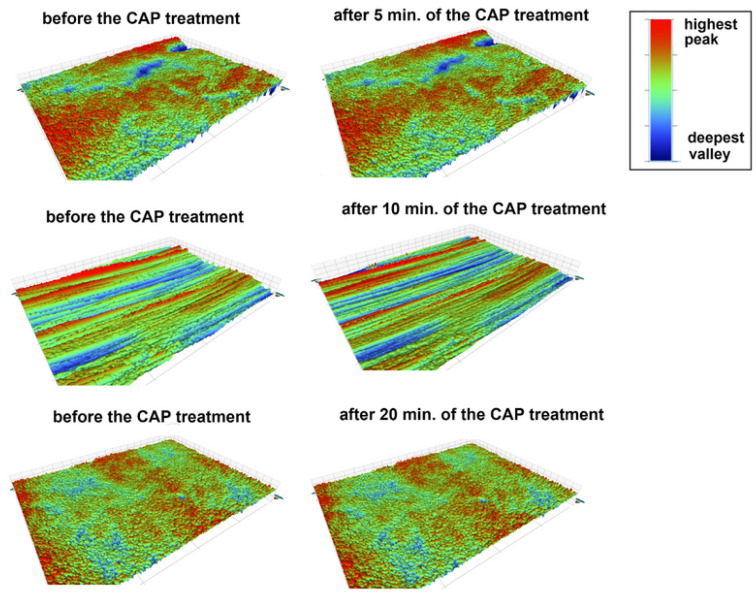
Images of the surface of the metal alloy (MA) before and after the CAP treatment (5, 10, 20 min), C-control/untreated sample (the colors represent the topography of the surface; the higher the peaks—the more red, the deepest the valleys—the more navy blue).

Table 2 presents the surface roughness parameters of the studied materials before and after the CAP treatment. The main roughness parameters: average roughness (R_a_), root-mean-square roughness (R_q_), peak-to-valley difference (R_t_) and the value of the largest profile depression (R_v_) were measured. As can be seen, the surface properties of acrylic changed during the plasma treatment. The R_a_ and R_q_ parameters did not change, but the surface after the plasma treatment exhibited a greater difference between peaks and valleys (higher R_t_ values) and deeper valleys (more negative R_v_ values). This observation was confirmed by the results presented in Figure 5. A comparison of the surface profiles at the same points before and after the plasma treatment clearly indicated the simultaneous appearance of higher and lower areas on the surface (intensification of red and blue colors), which was consistent with the data obtained in the SEM micrographs. In the case of the other two materials, i.e., acetal and prosthetic metal alloy, no differences in their surface roughness were observed. The differences in the roughness parameters were insignificant. These observations were also in accordance with the obtained 3D profiles of the material surfaces before and after the plasma treatment (Figure 5, Figure 6 and Figure 7) and the SEM measurement results.

#### 3.1.3. Changes in the Wetting Properties of the CAP-Treated Surfaces of the Prosthetic Materials

**Figure 8:**
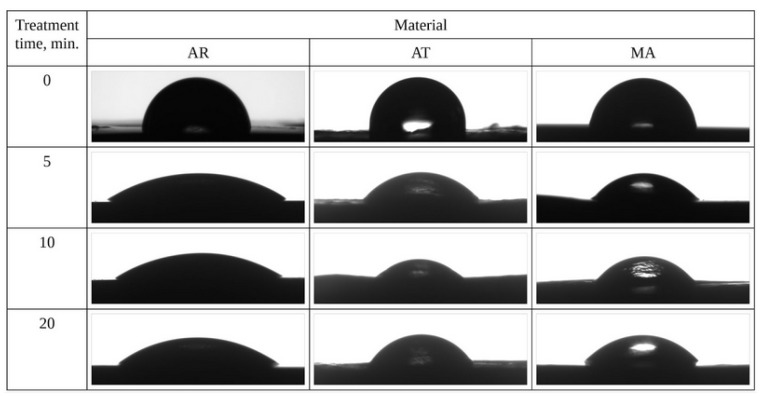
Images of water droplets on the surface of the materials before and after the plasma treatment.

**Figure 9:**
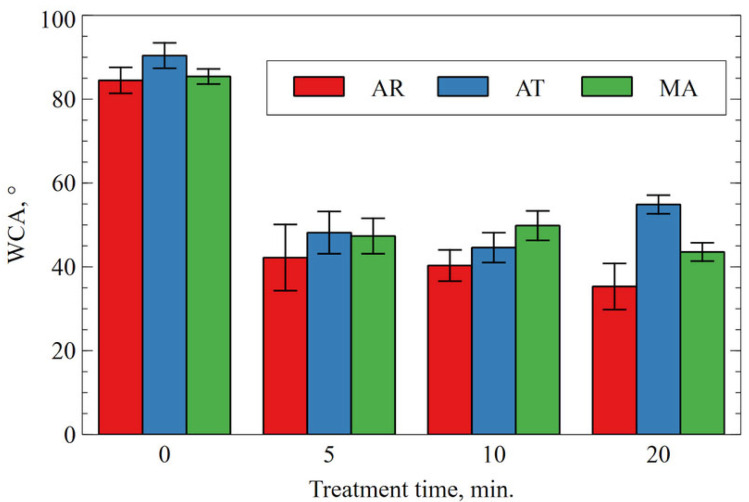
Average values of the contact angle of the material surfaces before and after the plasma treatment.

Images of droplets on the surface of the materials before and after the plasma treatment are shown in Figure 8. As in the previous studies using the tested reactor and helium as the main working gas [41,42], the treatment caused a reduction in the water contact angle. For all the processing times and materials, the contact angle decreased by at least 40%, with the effect being most visible for acrylic (50%, 52%, and 58% for 5, 10, and 20 min of treatment, respectively). The results between the individual plasma treatment times were usually within the error limit determined as a standard deviation, which could be related to both the relatively long treatment times and the differences on the surface of the control samples themselves (Figure 9). The lack of further significant variation in the contact angle after 5 min of treatment suggests that the surface reached a modification threshold, implying that this exposure time may represent the optimal duration for altering this parameter [43].

Reductions in the contact angle after treatment of similar materials have also been observed by other research groups. Vukušić et al. studied the effect of low-pressure oxygen plasma on acryl-coated polypropylene foils for food packaging, indicating that reducing the angle below 40 degrees can be difficult and causes etching of the material, worsening its functionality, in contrast to mild treatment, where the change in the contact angle occurred as a result of surface functionalization [44]. Guschl et al. studied the effect of radiofrequency plasma treatment of an acetal copolymer using a mixture of helium with a small amount of oxygen, after which an increase in surface energy (and thus a decrease in the contact angle) was observed. It was associated with etching or chain scission of the polymer [45]. The results obtained by Terpiłowski et al. showed that the reduction in the contact angle of metal alloys depended on their composition, but a reduction in all of them was observed after using a low-pressure reactor with argon or air as the working gas [46]. The observed effect was linked to modifications in surface topography and polarity, specifically due to the incorporation of polar oxygen-containing functional groups onto the material surface.

#### 3.1.4. Changes in the Color of the CAP-Treated Surfaces of the Prosthetic Materials

Spectrophotometric measurements on the CIE Lab scale are a precise method for assessing the color of dental materials. This method ensures color consistency, which is important for the aesthetics of prosthetic restorations and dental reconstructions. The AR-C control sample was characterized by an initial color lightness level L* (before the plasma treatment) of 36.86. The 5 min cold-plasma treatment increased the average value to 38.19, which indicates a brightening of the material surface. The CAP treatment of the acrylic plate for 10 min led to a further brightening of the surface, suggesting an intensification of the process and clear changes in its optical properties. On the other hand, prolonging the plasma exposure to 20 min led to a partial darkening of the sample relative to its condition at 10 min, potentially reflecting either the stabilization of plasma-induced surface effects or continued structural alterations at the surface level (Table 3).

The initial value of the L* parameter of 59.66 for the AT-C control sample gradually decreased with the extension of the CAP treatment time. The systematic decrease in the L* parameter suggested that the plasma affected the acetal surface structure, leading to its darkening. Possible mechanisms of these changes include oxidation and/or changes in reflective properties. The initial value of 53.09, corresponding to the MA-C control sample, increased slightly to 53.69 after the 5 min plasma treatment. The CAP treatment of the sample for 10 min caused the value of the described parameter to decrease to 52.47, which could indicate increased light absorption by the surface or the formation of new layers with reduced reflectivity. In turn, the increase in the MA-20 brightness to 65.17 could indicate significant surface transformations of the sample. A comparable enhancement of brightness upon plasma surface modification has been observed in ITO layers, where plasma treatment led to markedly improved luminance performance in OLED devices [47]. This could be the effect of oxidation and changes in reflective properties [48]. Such a significant change suggested that the longer exposure to plasma induced more intensive chemical and physical processes, such as removal of lower reflective surface layers, exposing more optically active regions of the material, reorganization of the surface structure leading to increased light reflectivity, and intensification of oxidation processes resulting in the formation of layers with high reflectivity. The data presented in Table 3 also show the effect of the plasma process on the value of the a* parameter of the three tested materials. AR showed dynamic changes in this value, which suggested surface modifications under the influence of CAP. After 5 min, a small increase in the parameter value was observed (from 8.25 to 8.38); however, after 10 min, there was a clear decrease in the chromaticity index. After 20 min, the value increased again to 8.48, which could indicate the reconstruction of the surface layer or stabilization of new optical properties of the material. The acetal plate was characterized by a relatively stable course of changes in the a* value during the first 10 min of plasma exposure (from 0.97 to 0.86). A significant decrease to 0.64 was observed only after 20 min, which could suggest slight degradation of the surface or changes in the chemical structure of the material. In turn, the metal-alloy plate showed a clearly different characteristic of changes in the a* value. During the first 10 min, the parameter value increased (from 0.66 to 0.95), which could be the effect of oxidation processes or adsorption of plasma molecules on the material surface. The decrease in the parameter to 0.18 in the MA-20 sample may indicate potential removal of the formed surface layers as a result of further plasma interaction.

The b* color parameter tests showed significant differences in the chromatic characteristics, which can have a significant impact on the visual properties of the analyzed materials (Table 3). The b* parameter value reflects a shift in the color towards yellow (for positive values) or blue (for negative values), which allows determining the dominant color component. In the samples from the AR group, small deviations of the parameter values were observed, except for the sample exposed to plasma for 10 min, where a clear shift towards blue color occurred (−2.16). The other samples from this group demonstrated relative color neutrality. Materials made of acetal were characterized by positive values of the parameter (dominance of a yellowish shade). The greatest shift towards yellow was observed in the AT-5 sample (6.04), while AT-20 was characterized by a lower intensity of this shade (4.59), which may indicate differences in the surface structure or the material composition. All the samples of the prosthetic metal alloy had negative b* values, corresponding to the dominance of cool, bluish tones. A particularly pronounced shift towards blue occurred in the material treated with a plasma jet for 20 min (−6.83). The effect of CAP on the color could be an effect of chemical reactions occurring on the surface, such as oxidation or changes in the structure. Plasma electrolytic oxidation (PEO) is an advanced technique for modifying metal surfaces, in which the application of high voltage in the electrolyte leads to the initiation of electric discharges generating plasma. This process induces metal oxidation, resulting in the formation of an oxide coating with diverse physicochemical properties. Process parameters, such as electrolytic composition, treatment time, and intensity, determine the structure, thickness, and optical properties of the formed layer, which can significantly affect the chromatic characteristics of the surface [49,50]. Plasma treatment with different plasma gases (argon, oxygen, and air), significantly modified the hydrophobic properties of such materials as polystyrene [51] or polycarbonate and polymethyl methacrylate [52], potentially affecting their optical characteristics. This process led to the introduction of oxygen-containing functional groups, which resulted in increased hydrophilicity of the material, as evidenced by reduced contact angles, also observed in our work. The color change in plastics could also be the effect of modifying their chemical structure and absorption properties [53,54]. As indicated by Bhatt et al. [55] and Wang et al. [56], plasma treatment improved adhesion by modifying the surface of materials, increasing their surface energy and wettability and introducing new functional groups.

### 3.2. Influence of CAP on the 12 h Fungal Adhesion to the Prosthetic Materials

**Figure 10:**
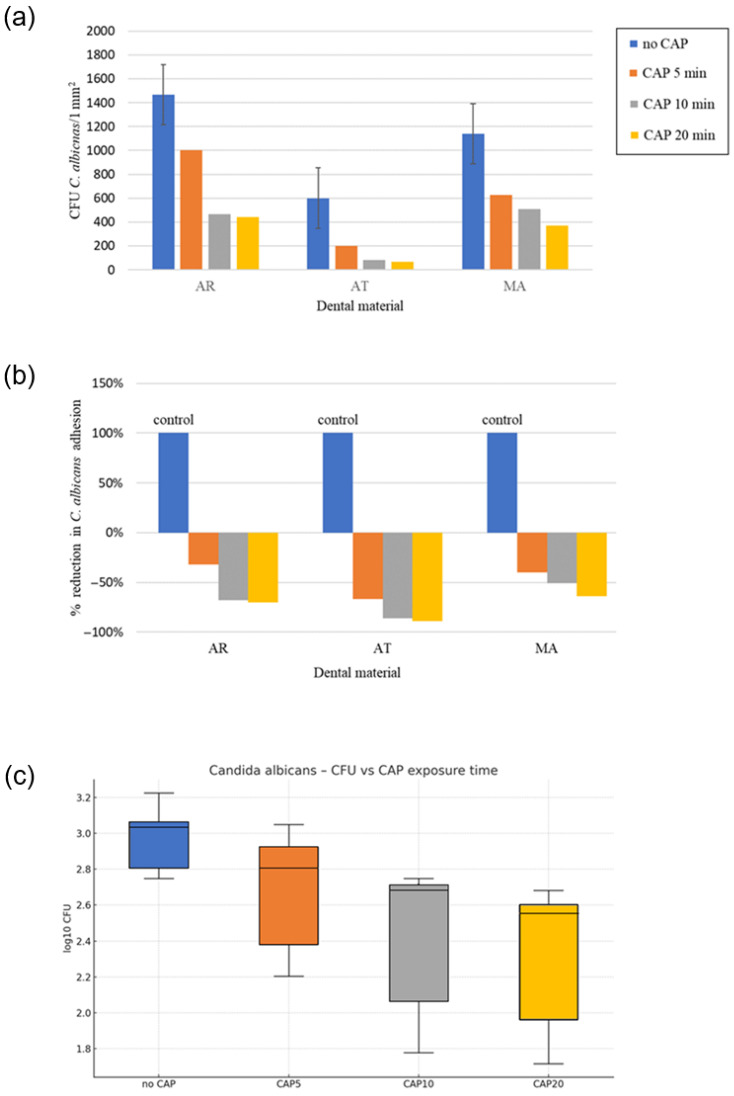
Effect of plasma treatment on 12 h adhesion of *C. albicans* ATCC to denture base materials (AR = acrylic resin; AT = acetal resin; MA = metal alloy) following standardized cold atmospheric plasma (CAP) exposure (5, 10, and 20 min). Control (no CAP) refers to the corresponding dental material samples not exposed to CAP: (**a**) Mean colony-forming units (CFU) of *C. albicans* per 1 mm^2^ after the CAP exposure; (**b**) Mean percentage reduction relative to the unexposed control; (**c**) Box-and-whisker distribution of log_10_ CFU for *C. albicans* versus the CAP exposure time. Control = material not exposed to CAP; bars show (**a**) mean CFU and (**b**) mean % reduction; (**c**) the box-plot depicts log_10_ CFU for *C. albicans* as a function of the CAP exposure time.

**Figure 11:**
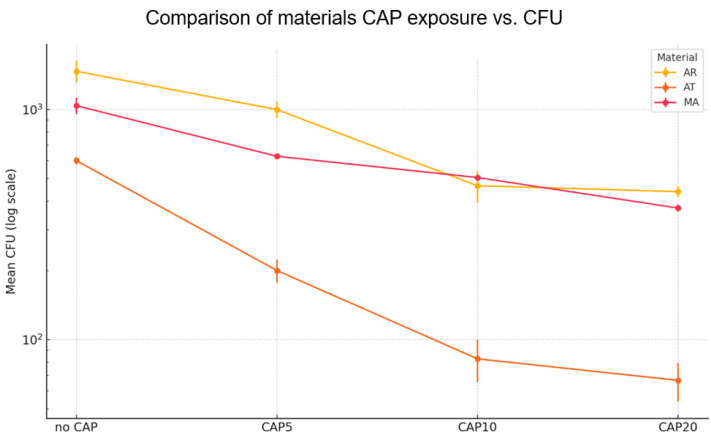
Material-specific response of *C. albicans* to the CAP exposure: mean ± sem log_10_ CFU plotted for acrylic resin (AR), acetal resin (AT), and metal alloy (MA) across the four treatment times.

**Figure 12:**
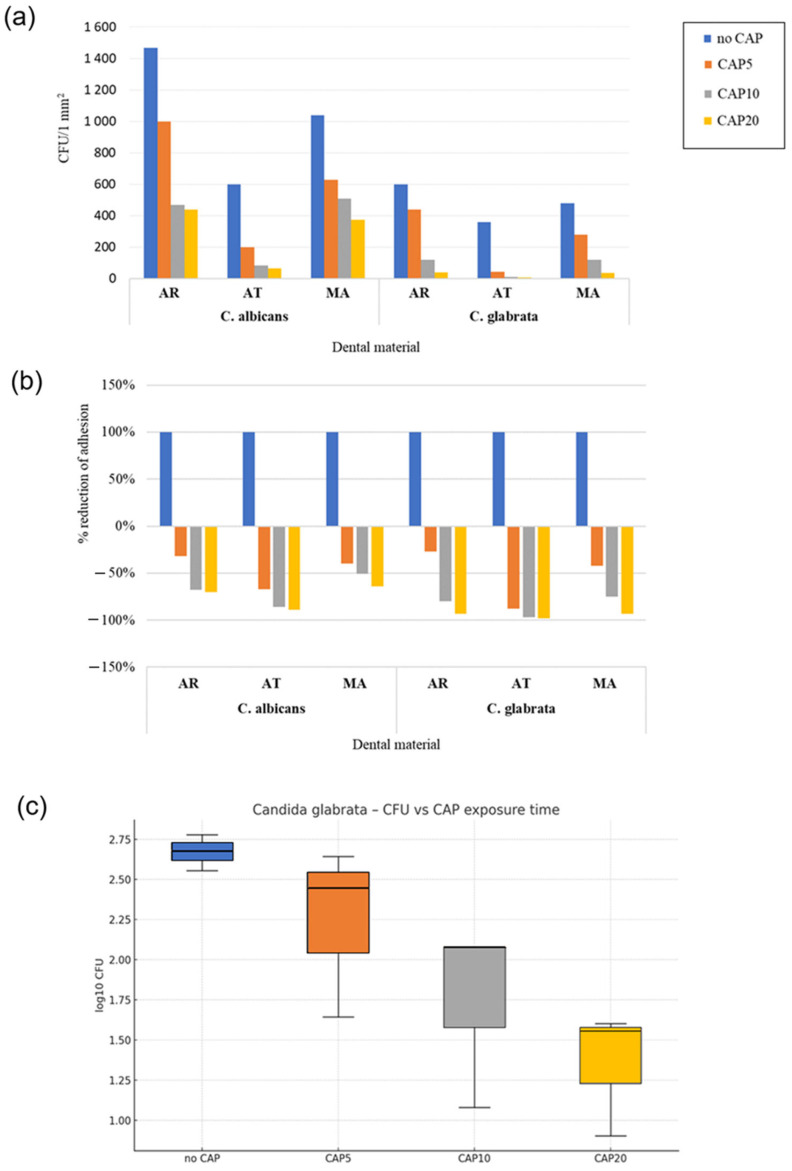
Comparison between the effect of the plasma treatment on the 12 h adhesion of single species *C. albicans* ATCC 10231 and *C. glabrata* ATCC 90030 to the prosthetic materials (AR—acrylic resin; AT—acetal resin; MA—metal alloy) following standardized cold atmospheric plasma (CAP) exposure (5, 10, and 20 min). Control (no CAP) refers to the corresponding dental material samples not exposed to CAP: (**a**) Mean colony-forming unit (CFU) count of *C. albicans* and *C. glabrata* per 1 mm^2^ of different dental materials; (**b**) Mean percentage reduction in *C. albicans* and *C. glabrata* growth (%) on three different dental materials; (**c**) Effect of the CAP exposure time on log_10_ CFU of *C. glabrata* ATCC 90030 adhering to the denture base resins. The box-and-whisker plot shows the distribution of log_10_ CFU at each exposure point.

In order to investigate the effect of the plasma treatment on the adhesion of denture stomatitis-causing fungi to prosthetic materials, a set of microbiological studies involving both *C. albicans* (pseudo/hypha producer, azole-susceptible species) and *C. glabrata* (non-pseudo/hypha producer, azole-resistant species) was carried out (Figure 10, Figure 11 and Figure 12).

#### 3.2.1. Effect of CAP on 12 h Adhesion of Reference *C. albicans* to the Prosthetic Materials

A significant decrease in *C. albicans* adhesion (expressed as CFU/1 mm^2^) was observed following the CAP treatment, with the strongest reduction recorded on AT ≈ 89% at 20 min (Table 4; Figure 10b). CAP decreased the 12 h adhesion of *C. albicans* on every denture resin (Figure 10c), a result which supports the current large body of literature describing ≥ 1 log antimicrobial effects within clinically relevant exposure times [57,58].

The acetal resin (AT) was characterized by smoother baseline topography, and the greater polar component of surface free energy seems to enhance the hydrophilization generated by CAP, thus generating conditions unfavorable for the initial *Candida* cell attachment, which accords with earlier findings that AT is more sensitive to short-term plasma functionalization than highly cross-linked polymethyl methacrylate (AR) [58]. In contrast, the metal alloy (MA) exhibited the lowest absolute decrease in the adhesion of fungi to the denture base materials (Figure 10, Figure 11 and Figure 12), However, even on MA, the log transformed counts decreased by more than 0.5 following the 10 min protocol (Figure 11).

A two-way ANOVA of log_10_–transformed colony-forming units (CFU mm^−2^) showed significant main effects of the material (η^2^ = 0.53, ω^2^ = 0.52), CAP exposure time (−η^2^ = 0.38, ω^2^ = 0.38), and material × time interaction (η^2^ = 0.06, ω^2^ = 0.05). While the material × time interaction was statistically significant in the two-way ANOVA, its effect size was minimal (ω^2^ ≈ 0.05). Practically, this implies that the downward trend in CFU was monotonic for all the resins, and one 10 min setting is likely to be sufficient across the range of materials routinely used for removable prostheses. The extension of the CAP exposure from 10 to 20 min did not lead to any further significant reduction in the 12 h adhesion of *C. albicans* on any of the tested materials (Figure 11). Tukey HSD post-hoc comparisons confirmed that every CAP treatment (5, 10, 20 min) reduced adhesion versus the unexposed control (*p* < 0.01) and that the 10 min and 20 min variants did not differ from each other (*p* > 0.05). Prolonging the CAP exposure to 20 min achieved only incremental additional killing (<3% compared to 10 min) and did not enhance any of the physicochemical parameters assayed in parallel (roughness, wettability, color stability). The lack of a clinically significant advantage beyond 10 min is consistent with the kinetic plateau described by Trebulova [59] and is promising from a chairside perspective, since extended treatment times would detract from workflow and patient comfort without proportional increases in efficacy. The degree of response was a function of both the type of material and the plasma treatment time, but a single 10 min cycle in all the instances was sufficient to produce a biologically significant reduction in colony forming units of *C. albicans*.

#### 3.2.2. Effect of CAP on 12 h Adhesion of Reference *C. glabrata* to the Prosthetic Materials

Similarly, the acetal resin (AT) CAP treatment resulted in the steepest decline in the 12 h adhesion of *C. glabrata* (≈98% at 20 min) (Figure 12, Table 5). The ≥10-min CAP treatment produced ≥75% reduction across all the resins. *C. glabrata* exhibited lower baseline adhesion compared to *C. albicans* on all the tested dental materials prior to the CAP exposure (Figure 12a,b). The lower baseline adhesion and higher percentage reduction in adhesion observed for *C. glabrata* is likely related to its morphological characteristics, as it produces only round blastospores in contrast to *C. albicans*, which is capable of forming both blastospores and filamentous hyphae. The supporting experiment with *C. glabrata*, which is inherently less susceptible to azole treatment, strengthened these findings. Unfortunately, only a single measurement was available for each material × time combination. We pooled the three materials and performed a one-way ANOVA on log_10_ CFU by the CAP exposure time (per level). The effect of the CAP exposure time was significant (strong impact: η^2^ = 0.66, ω^2^ = 0.51) (Figure 12c). The one-way ANOVA on log_10_ CFU revealed a robust time-dependent decrease (η^2^ = 0.66), once more plateauing after 10 min. Although the percentage reduction continued to rise numerically up to 20 min, the additional gain was ≤ 18 percentage points and therefore clinically marginal (Figure 10a–c).

The more pronounced slope on the studied materials might be attributed not merely to the surface chemistry but also to the thinner *β* glucan layer of *C. glabrata*, which may have allowed deeper penetration of reactive oxygen and nitrogen species formed in the plasma [59,60]. Notably, the rank order of susceptibility among the materials was identical to that of *C. albicans*, implying that a common protocol can be viable even for mixed biofilms.

Based on the presented results, it is clearly visible that the plasma-based technology reduced early biofilm growth of reference *Candida* species on all the tested material surfaces. The 10 min CAP exposure was sufficient to significantly reduce the 12 h adhesion of the single species *C. albicans* and *C. glabrata.* Cumulatively, these results endorse the implementation of a 10 min helium jet CAP cycle as a practical adjunct to denture hygiene. This quick and non-thermal treatment seems to preserve the optical and mechanical integrity of the polymer whilst conferring a significant antifungal advantage. This 65% threshold was used as a pragmatic cut-off for clinically relevant early biofilm control, echoing reports that a two-third reduction in denture biofilm is associated with symptomatic relief [57,61].

## 4. Conclusions

Cold atmospheric plasma (CAP) is a rapid non-thermal adjunct to denture base decontamination. One 10 min helium-jet cycle reduced *Candida* loads on all the resins by ≥ 1 log, with large effect sizes for both the material factor (η^2^ ≈ 0.53) and the exposure time (η^2^ ≈ 0.38). Prolonging the treatment to 20 min yielded only marginal further killing (<3%), demonstrating a distinct efficacy plateau.

In both *Candida* species, the AT resin was most responsive, reaching up to 98 % adhesion reduction for *C. albicans* and 97–98% for *C. glabrata*, while the traditional AR resin exhibited the lowest but still clinically significant reduction. The same hierarchy of material susceptibility and the strong time-dependent response found for *C. glabrata* (η^2^ = 0.66) suggest that the same 10 min protocol can be used confidently on mixed *Candida* biofilms. One 10 min CAP cycle decreased *C. albicans* and *C. glabrata* loads on the conventional denture base resins by at least one logarithmic unit. This study demonstrates that cold atmospheric plasma (CAP) treatment holds promise as a method for maintaining the hygiene of prosthetic materials. CAP was found to be chemically safe for all the acrylic, acetal, and metal-alloy materials, without significantly altering their elemental composition. However, distinct material-specific responses were observed under the CAP treatment. The acetal resin and the metal alloy exhibited stability in surface morphology, roughness, and color following the CAP exposure together with the ability to reduce the water contact angle and potential fungal adhesion. This supports the use of CAP for cleaning and decontaminating these materials. In turn, the acrylic resin showed substantial topographical and optical changes, including increased surface roughness and altered brightness. These modifications may compromise its structural integrity and aesthetic qualities, making CAP unsuitable for acrylic-based prostheses in clinical use. Overall, CAP treatment emerges as a promising adjunctive technology for enhancing prosthetic hygiene and reducing *Candida albicans* and *Candida glabrata* colonization, provided material-specific limitations are considered. Plasma treatment can be used to clean and activate surfaces of certain materials by removing adsorbed contaminants and introducing polar functional groups (e.g., hydroxyl, carbonyl, or carboxyl groups). As the population ages, denture stomatitis prevalence will increase; therefore, there is an increasing demand for new preventive strategies, and the current results offer an encouraging proof of principle.

Limitations. The in vitro study used a static 12 h fungal adhesion model, capturing early biofilm growth, using single-species reference strains and investigated only one CAP parameter setting (helium jet, 25 kHz, 7 W). Since only early biofilm growth was assessed and neither mature nor mixed-species biofilms were tested, the anti-adhesion/anti-biofilm effect of CAP may be overestimated relative to more complex, clinically relevant conditions. Another partial limitation of the study is that only 2D roughness parameters were analyzed. According to ISO 25178 [62], 3D areal parameters and sampling from clinically relevant fields would be necessary for a more representative characterization of prosthetic surfaces.

Future work. Subsequent research should address mixed, dual, and multispecies dynamic biofilms in salivary-pellicle conditions and optimize plasma parameters (gas composition, power, and exposure time) to maximize antifungal efficacy in prosthetics in conditions resembling those in the oral cavity. Incorporation of CAP application into clinical workflow to prevent denture stomatitis caused by yeast overgrowth remains a challenge.

## Figures and Tables

**Table 1:** Surface composition (wt.%) of the prosthetic materials before and after cold atmospheric plasma treatment: C-control; 5 min treatment; 10 min treatment, and 20 min treatment, (mean ± SD).

	AR-C [wt.%]	AR-5 [wt.%]	AR-10 [wt.%]	AR-20 [wt.%]
C	65.17 ± 0.78	64.93 ± 0.78	65.82 ± 0.82	66.56 ± 0.91
O	34.83 ± 0.77	35.07 ± 0.73	34.18 ± 0.85	33.44 ± 0.81
	**AT-C [wt.%]**	**AT-5 [wt.%]**	**AT-10 [wt.%]**	**AT-20 [wt.%]**
C	46.95 ± 0.46	47.47 ± 0.49	47.74 ± 0.86	46.59 ± 0.53
O	53.05 ± 0.53	52.53 ± 0.41	52.26 ± 0.78	53.41 ± 0.67
	**MA-C [wt.%]**	**MA-5 [wt.%]**	**MA-10 [wt.%]**	**MA-20 [wt.%]**
Co	49.69 ± 0.72	49.78 ± 0.61	50.14 ± 0.62	50.07 ± 0.63
Cr	24.47 ± 0.39	24.53 ± 0.42	24.27 ± 0.35	24.82 ± 0.48
O	9.72 ± 0.76	9.96 ± 0.45	9.77 ± 0.52	9.69 ± 0.64
C	6.55 ± 0.65	5.54 ± 0.45	5.55 ± 0.44	5.29 ± 0.31
Mo	4.06 ± 0.08	4.06 ± 0.01	4.46 ± 0.47	4.07 ± 0.04
Si	3.14 ± 0.09	3.22 ± 0.06	3.53 ± 0.07	3.18 ± 0.03
Al	1.73 ± 0.27	2.21 ± 0.34	1.54 ± 0.12	1.89 ± 0.13
Fe	0.64 ± 0.04	0.70 ± 0.01	0.74 ± 0.05	0.69 ± 0.06

**Table 2:** Roughness parameters of the studied materials before and after the CAP treatment. SD ≤ 8%.

AR-5	before CAP	after 5 min of CAP
R_a_	1.06	1.08
R_q_	1.39	1.42
R_t_	22.62	40.41
R_v_	−15.23	−33.48
**AR-10**	**before CAP**	**after 10 min of CAP**
R_a_	0.71	0.73
R_q_	0.92	0.96
R_t_	26.27	43.03
R_v_	−20.84	−37.85
**AR-20**	**before CAP**	**after 20 min of CAP**
R_a_	1.76	1.82
R_q_	2.23	2.31
R_t_	23.46	32.47
R_v_	−15.85	−34.36
**AT-5**	**before CAP**	**after 5 min of CAP**
R_a_	5.69	5.63
R_q_	7.18	7.12
R_t_	57.31	56.36
R_v_	−30.13	−29.34
**AT-10**	**before CAP**	**after 10 min of CAP**
R_a_	4.23	4.17
R_q_	5.36	5.98
R_t_	47.93	47.52
R_v_	−18.72	−20.57
**AT-20**	**before CAP**	**after 20 min of CAP**
R_a_	5.32	5.27
R_q_	7.43	7.49
R_t_	77.99	76.17
R_v_	−32.75	−31.32
**MA-5**	**before CAP**	**after 5 min of CAP**
R_a_	0.53	0.42
R_q_	0.69	0.54
R_t_	9.18	8.38
R_v_	−5.88	−5.15
**MA-10**	**before CAP**	**after 10 min of CAP**
R_a_	1.75	1.82
R_q_	2.18	2.28
R_t_	19.87	20.03
R_v_	−7.51	−8.74
**MA-20**	**before CAP**	**after 20 min of CAP**
R_a_	0.42	0.41
R_q_	0.55	0.54
R_t_	8.86	8.83
R_v_	−5.10	−5.63

**Table 3:** Lab* parameter values for dental composite samples before and after the exposure to cold plasma for a specified time: C- control; 5 min treatment; 10 min treatment, and 20 min treatment (mean ± SD).

Color Coordinates	AR-C	AR-5	AR-10	AR-20
Brightness (*L*)	36.86 ± 0.29 ^a^	38.19 ± 0.35 ^b^	39.26 ± 0.50 ^c^	37.85 ± 0.10 ^b^
Chromatic component *a*	8.25 ± 0.07 ^b^	8.38 ± 0.18 ^b^	6.68 ± 0.32 ^a^	8.48 ± 0.27 ^b^
Chromatic component *b*	0.59 ± 0.09 ^b^	0.93 ± 0.13 ^c^	−2.16 ± 0.08 ^a^	0.56 ± 0.08 ^b^
	**AT-C**	**AT-5**	**AT-10**	**AT-20**
Brightness (*L*)	59.66 ± 0.49 ^b^	59.00 ± 0.20 ^ab^	58.91 ± 0.28 ^ab^	58.66 ± 0.03 ^a^
Chromatic component *a*	0.97 ± 0.03 ^b^	0.96 ± 0.06 ^b^	0.86 ± 0.03 ^b^	0.64 ± 0.14 ^a^
Chromatic component *b*	5.69 ± 0.11 ^b^	6.04 ± 0.19 ^c^	4.86 ± 0.37 ^ab^	4.59 ± 0.61 ^a^
	**MA-C**	**MA-5**	**MA-10**	**MA-20**
Brightness (*L*)	53.09 ± 0.80 ^a^	53.69 ± 1.08 ^a^	52.47 ± 1.02 ^a^	65.17 ± 0.89 ^b^
Chromatic component *a*	0.66 ± 0.12 ^b^	0.79 ± 0.21 ^b^	0.95 ± 0.09 ^b^	0.18 ± 0.01 ^a^
Chromatic component *b*	−5.45 ± 1.42 ^ab^	−4.81 ± 0.81 ^ab^	−3.86 ± 0.07 ^b^	−6.83 ± 0.06 ^a^

^a,b,c^—values in the rows marked with different letters are statistically significantly different at *p* ≤ 0.05.

**Table 4:** Data showing mean ± sem (sem—standard error of the mean) of CFU and mean % reduction in *C. albicans* ATCC 10231 adhesion vs. control.

Mean ± Sem CFU and %-Reduction vs. Control				
Material	Treatment	Mean CFU	Sem CFU	% Reduction
AR	no CAP	1467	157.2	0
AR	CAP5	1000	83.3	32
AR	CAP10	467	74.2	68
AR	CAP20	440	23.1	70
AT	no CAP	600	23.1	0
AT	CAP5	200	23.1	67
AT	CAP10	83	17	86
AT	CAP20	67	12.7	89
MA	no CAP	1040	83.3	0
MA	CAP5	627	13.3	40
MA	CAP10	507	13.3	51
MA	CAP20	373	13.3	64

**Table 5:** Data showing mean ± sem (sem—standard error of the mean; sem is not calculable when *n* = 1) of CFU and mean % reduction in *C. glabrata* ATCC 90030 adhesion vs. control.

Mean ± Sem CFU and %-Reduction vs. Control–*C. glabrata*				
Material	Treatment	Mean CFU	Sem	% Reduction
AR	no CAP	600	—	0
AR	CAP5	440	—	27
AR	CAP10	120	—	80
AR	CAP20	40	—	93
AT	no CAP	360	—	0
AT	CAP5	44	—	88
AT	CAP10	12	—	97
AT	CAP20	8	—	98
MA	no CAP	480	—	0
MA	CAP5	280	—	42
MA	CAP10	120	—	75
MA	CAP20	36	—	93

## Data Availability

The original contributions presented in this study are included in the article. Further inquiries can be directed to the corresponding author(s).
